# Morphological diversity and altitudinal differentiation of *Aethopyga* species

**DOI:** 10.1002/ece3.10473

**Published:** 2023-08-30

**Authors:** Wenzhu Lu, Shimiao Shao, Lingling Zu, Xu Luo, Yubao Duan

**Affiliations:** ^1^ Key Laboratory for Conserving Wildlife with Small Populations in Yunnan Southwest Forestry University Kunming China; ^2^ Faculty of Biodiversity Conservation Southwest Forestry University Kunming China; ^3^ State Key Laboratory for Diagnosis and Treatment of Infectious Diseases, The First Affiliated Hospital Zhejiang University School of Medicine Hangzhou China; ^4^ Key Laboratory for Forest Resources Conservation and Utilization in the Southwest Mountains of China, Ministry of Education Southwest Forestry University Kunming China; ^5^ Key Laboratory of Forest Disaster Warning and Control in Universities of Yunnan Province Southwest Forestry University Kunming China

**Keywords:** *Aethopyga*, altitude, distribution overlap, geometric morphometrics, morphology, phylogenetic relationship

## Abstract

The morphological characteristics of birds are an important tool for studying their adaptation and evolution. The morphological evolution of a clade is not only constrained by the phylogenetic relationship, but also influenced by ecological factors and interspecific competition. *Aethopyga* is a group of small nectar‐eating birds with obvious sexual dimorphism. They have slender and decurved beaks, which reflect their unique diet and foraging mode. Traditional and geometric morphometrics were combined to characterize the body morphology and beak shape of six species of *Aethopyga* distributed in China. We aim to assess the roles of phylogeny, altitude, and species interactions to morphological evolution. The main distinguishing characteristic among these six species were overall body size, the ratio of body weight, culmen and tarsal length to body length, tail length and wing length, and beak shape (slender/straight vs. thick/decurved). Although these dimensions cannot distinguish all species, they can show a clear distribution trend, and there is a significant Mahalanobis distance between each pair of species. There were no significant phylogenetic signals in morphological traits. The results of PGLS analysis show that altitude is significantly correlated with log‐transformed tarsus length and beak‐shaped PC1 (slender/straight vs thick/decurved dimensions) across the six species analyzed. Mantel test shows that the distance matrix of beak morphological characteristics showed a significant correlation with the altitudinal distance matrix. The results indicated no significant phylogenetic signal in the morphological characteristics of six species. In terms of beak shape, species with greater overlap in elevation distribution have more similar morphological characteristics, that is, less morphological differentiation.

## Graphical abstract

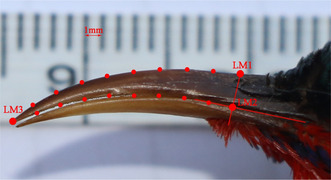

## 1 BACKGROUND

Morphological diversity is an important part of biodiversity. The phenotypic characteristics of species are the result of a variety of selective pressures, including migration (Marchetti et al., 1995), habitat selection (Zeffer et al., 2003), foraging behavior (Sonne et al., 2019), sexual selection (Evans, 2004; Price, 1991), and avoidance of natural enemies (Ge et al., 2011). The morphological diversity of biological groups is due to the directional selection of ecological factors on the one hand, and on the other hand, the result of ancestral developmental regulation genes and phylogenetically conserved traits (Burns et al., 2002). The beak of birds is a part that scholars have paid more attention to. Its shape is not only closely related to feeding habits and foraging behavior (Kulemeyer et al., 2009; Pigot et al., 2020), but also related to the use of visual sense organs, the ability to rearing chicks (Martin, 2007), and sexual selection (Derryberry et al., 2018; Huber & Podos, 2006). Environmental filtering hypothesis indicates that species occupy the same geographic region have adapted to similar vegetation, habitat and food types, and thus have evolved similar morphological characteristics (Keddy, 1992; Kraft et al., 2015). Altitude influences temperature, precipitation, and habitat types, which may in turn exert selective pressures on the morphological evolution of organisms through factors, such as food availability, shelter, and activity patterns, potentially promoting phenotypic evolution along elevation gradients (Barry, 2008; Kennedy et al., 2012; McCain & Grytnes, 2010; Price et al., 2014). Previous studies have shown that the relative size of the beak of different species is positively correlated with the temperature of the habitat environment (Danner et al., 2017). The body size also vary, both among related species and within a species, along an altitudinal gradient, as a result of its response to changes in temperature (Ashton, 2002; Mayr, 1956). On the other hand, the closer the relationship between two species is, the more likely they will have similar characteristics such as structure and behavior, and correspondingly have similar ecological needs, which will lead to competition (Smith, 1967). Exotic relatives are geographically isolated to reduce competition, while similar relatives of the same domain need to have differences in at least one aspect of habitat, food habits, and foraging methods to prevent overlapping needs and avoid competition, which can often be reflected in the differentiation of morphology (Price, 1991; Smith, 1967). Therefore, the two mechanisms of environmental filtering and sympatric competition may work together on the morphology of organisms.

*Aethopyga*, a genus of sunbirds, are small nectar‐eating birds with obvious sexual dimorphism. The male plumage is brightly colored and the feathers are mostly metallic, as an adaptation for sexual signaling, while the female plumage is relatively plain, mostly olive green or grayish brown. *Aethopyga* usually stop on plants when they feed on nectar. Similar to other nectar‐eating birds, the beak of *Aethopyga* species is narrow and downwardly curved, and their morphology presents a distinct sexual dimorphism, and males usually have heavier weight, longer wings, and longer beaks (Collins & Paton, 1989; Paton & Collins, 1989). There are 18 species in *Aethopyga*, making it the second largest genus of the Nectariniidae (Cheke & Mann, 2008), mainly distributed in the Indian subcontinent, Southeast Asia, Indochina, and the Philippines. This genus of birds has two major distribution centers, the Philippine Islands (eight species), and the Himalayan‐Hengduan Mountains region (six species) (Cheke & Mann, 2008). *Aethopyga* sunbirds have well elucidated phylogenetic relationships (Hosner et al., 2013), making them a roubst object for exploring morphological adaptation from an evolutionary perspective. The literature and previous field survey results show that there are five species of *Aethopyga* in the Gaoligong Mountains in the Himalayan‐Hengduan Mountains region, and they are distributed in an altitude gradient from the foot to the top of the mountain (140–4000 m above sea level) which provides a favorable research system for adaptive evolution of birds at an altitude gradient and the competition and interaction mechanism of species in the same region.

Morphological research needs to effectively extract and analyze biological size and shape information. Morphometrics measure variables, such as length, width, and height, and further compare the means or use multivariate statistics methods to quantitatively extract and analyze information related to the size and shape of organisms (Slice, 2007), so as to compare the differences and similarities between different groups (Rohlf & Marcus, 1993). In comparison, geometric morphology selects biologically significant landmarks of the research object to represent the shape information of biological structures, which can remove the influence of size on shape by overprinting, fully retain comprehensive shape information (Adams et al., 2004). Therefore, we combined traditional morphometrics and geometric morphometric, through which we attempt to investigate the effect of phylogenetic relationships and elevation on species morphology.

## 2 METHODS

### 2.1 Data collection

We measured a total of 298 museum specimens belonging to six species (Table S1) from the Southwest Forestry University Specimens Museum, The Specimens Museum of Kunming Institute of Zoology of the Chinese Academy of Sciences, and the National Animal Specimen Resource Bank of the Institute of Zoology, Chinese Academy of Sciences. Only specimens of male adult individuals were measured. Body weight and body length were obtained from the original field records taken during field collection. Wing length, tail length, tarsus length, and culmen length were measured with an accuracy of 0.1 mm using a digital caliper (Table S2 and Appendix S2). In addition, the images of the beaks of 298 specimens were taken using Canon EOS 70D camera for geometric morphometric analyses. In our study, we followed a standardized protocol to ensure consistent distance and angle during the measurements. A tripod was used and both the camera and the subject were placed in marked spots and positioned in a standardized manner, ensuring a consistent distance and angle between the camera and the specimen for all photographs taken. Subsequently, we identified specific anatomical landmarks on the subjects (e.g., bill tip) to measure the desired morphological traits consistently across all photographs. By implementing these measures, we aimed to minimize any potential biases introduced by variations in photo angle and ensure reliable and comparable measurements throughout our study.

### 2.2 Body morphology

All measurements were log‐transformed for normalized distributions before statistical analyses (Kaboli et al., 2007). One‐way ANOVAs were performed to compare each character among the species. In addition, a canonical variate analysis (CVA) was conducted on the data of all individuals to extract axes with most interspecific difference and to generate a matrix of pairwise Mahalanobis distances based on the six log‐transformed variables (Campbell & Atchley, 1981).

Before further analyses, all data were standardized to a mean of zero and a variance of one to reduce the effect of different units of measure. Subsequently, a principal component analysis (PCA) was applied to species mean values of each linear measurement to extract and visualize major variation. To preserve the information of body size, which contains important evolutionary and ecological indications (Alatalo et al., 1986), the data were not size‐corrected before the PCA. The statistical analyses were performed with PAST 2.17 software (Hammer et al., 2001).

### 2.3 Beak shape variation

**FIGURE 1:**
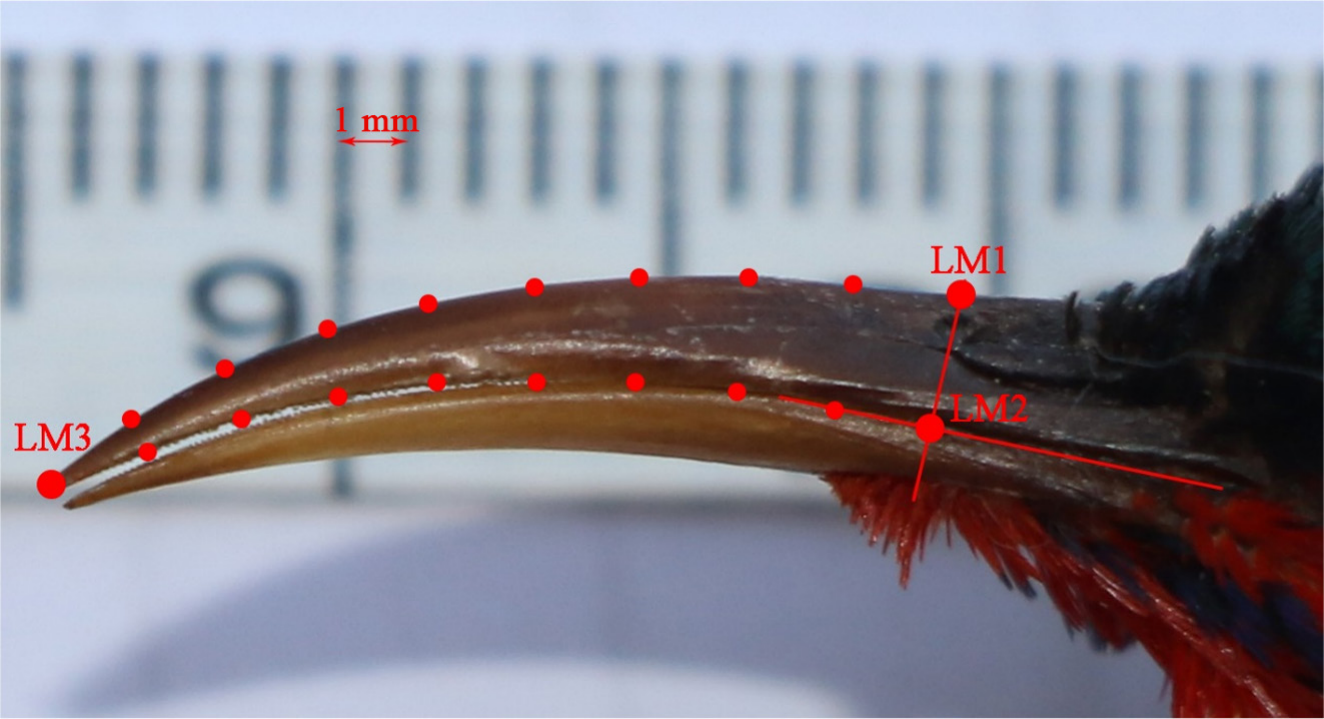
Landmarks and semi‐landmarks used for the geometric morphometric analysis. A line perpendicular to the suture was drawn across the rostral edge of the nares. Two landmarks were placed where this line intersects the outline of the upper mandible, whereas the third was placed at the tip of the beak. Eight semi‐landmarks were placed equidistantly between LM1 and LM3, and the other eight between LM2 and LM3.

To describe the shape and characteristics of the beak, eight equidistant semi‐landmarks were set on the upper and lower curves of the upper beak. We used tpsUtil (Rohlf, 2009) software to create an executable file in tps format from the picture, tpsDig (Rohlf, 2005) software to mark all landmarks, and tpsRelw (Rohlf, 2003) to slide the semi‐landmarks. Subsequently, the aligned specimens (Appendix S3) were imported into MorphoJ (Klingenberg, 2011) software for morphological variation analysis. In this study, we selected the tip of the beak as the first landmark. Due to the lack of homologous landmarks on bird beaks and the extensive coverage of feathers and rictal bristles at the base of the beak in the *Aethopyga*, it was not possible to obtain a clear image of the entire beak. Therefore, a line perpendicular to the commissure of the beak was drawn through the anterior edge of the nostril using AutoCAD, and the intersection of this line with the beak was marked as the landmark (see Figure 1).

Procrustes superimposition was applied to eliminate non‐shape variation, such as size and orientation (Rohlf, 1999; Slice, 2001). The CVA was then performed on all configurations to identify the dimensions that best discriminating among species and generate a matrix of pairwise Mahalanobis distances (Campbell & Atchley, 1981). In addition, a Procrustes ANOVA (Klingenberg et al., 2002) was conducted to describe general interspecific difference. The PCA was performed on species mean shapes to extract dimensions with most of the variation in beak shape.

### 2.4 Phylogenetic and comparative approach

We utilized the Maximum Likelihood approach to construct the phylogenetic tree. Specifically, we employed RAxML (Stamatakis et al., 2005) with gene partitions. The branch lengths were then calculated using three nuclear genes (Beta‐Fibrinogen intron 5, Transforming growth factor beta‐2 intron 5 and Z‐linked muscle skeletal receptor tyrosine kinase) and two mitochondrial genes (NADH dehydrogase‐2 and NADH dehydrogenase‐3). In our study, the sequences used for constructing the phylogenetic tree were obtained from GenBank (Table S3). The accession numbers for these sequences can be found in the study by Hosner et al. (2013). The best‐fit models for the analysis were selected based on the Bayesian Information Criterion (BIC). The selected models included the general time‐reversible model with gamma‐distributed rates among sites and invariant sites (GTR + I + G) for the 1st and 3rd mitochondrial positions, and the HKY + I + G model for the 2nd position. For the nuclear intron Fib‐5, the HKY model was chosen, the HKY + G model was selected for TGFβ5, and the HKY + I + G model was utilized for MUSK.

Then we projected the phylogenetic tree reconstructed (Appendix S4) onto the shape spaces of the PC scores computed from the mean body morphology and mean beak shape to visualize the trajectories of morphological evolution. The squared‐change parsimony method was used to infer the ancestral state of each internal node. (Maddison, 1991).

Blomberg's *K* and Pagel's *λ* were used to assess the phylogenetic signal in morphological traits (Blomberg et al., 2003; Pagel, 1999). A value near 0 for both indices suggests that the traits are evolving independently of the phylogenetic relationships. On the contrary, a value of 1 suggests that the traits are evolving according to the Brownian‐motion model, while values that are higher than 1 suggest that traits among related species are more similar than expected under a Brownian‐motion model (Blomberg et al., 2003; Freckleton et al., 2002). The effect of body size was accounted by using the residuals of regressions of linear traits on body weight, both log‐transformed (Price et al., 2014). Phylogenetic signal was tested in log‐transformed linear traits, size‐corrected traits, and PC values generated from body morphology and beak shape.

### 2.5 Analysis of the pattern and formation mechanism of the morphological diversity along altitude gradients

Regression analyses were carried out to check for potential covariation between morphological traits and altitudinal distribution, both interspecifically and intraspecifically. For interspecific covariation, a phylogenetic generalized least square (PGLS) regression was performed, which regresses species mean values of each morphological trait on the medium of altitudinal ranges of species while correcting for phylogenetic relationships (Mundry, 2014). For intraspecific covariation, we conducted a regression of each morphological trait on the altitude of all individuals belonging to *A. nipalensis*, a species occupying a wide altitudinal range (1280–4000 m). Regressions were conducted with log‐transformed traits and size‐corrected traits (residuals of regressions of log‐transformed measurements on log‐transformed body weight) to correct for the potential effect of body size. All elevation data were collected from the original record of the sampling site for each specimen.

In addition, we analyzed the relationship between the degree of altitudinal overlap and morphological differentiation among all species. The degree of altitudinal overlap between two species was calculated as follows: DO = OAB/(EA + EB − OAB), where EA and EB are the altitude range of two species, and OAB is the overlap of the two species. Then DAB = 1 − DO was defined as the altitude distribution difference distance index, and subsequently a pairwise matrix of the altitude distribution distance was generated. The Mahalanobis distance matrices of body morphology and beak shape obtained by canonical variable analysis (CVA) were used as morphological distances.

We conducted a partial Mantel test (Mantel, 1967) (10,000 permutations) to investigate whether there is correlation between character differentiation and distributional overlap between species. The matrices of morphological distances were compared with the matrix of distributional distance, and phylogenetic distances were included to account for phylogenetic relatedness.

PAST 2.17 (Hammer et al., 2001) was used to perform the PCA, ANOVA, ANCOVA, CVA of linear measurements and regression analysis, and MorphoJ (Klingenberg, 2011) was used to conduct the geometric morphometric analysis for beak shape variation. We used the package ape (Paradis et al., 2004), geiger (Harmon et al., 2008), and phytools (Revell, 2012) in R (R Core Team, 2022) (R scripts see Appendix S5) for phylogenetic signal test, the package nlme (Pinheiro et al., 2015) in R for the PGLS analysis, and the package ncf (Bjornstad & Cai, 2020) in R for the Mantel test. Since *A. nipalensis* and other species have less overlap, two datasets, one including all six species and the other excluding *A. nipalensis*, were used to conduct phylogenetic signal and Mantel tests.

## 3 RESULTS

### 3.1 Variation in body morphology

Significant interspecific differences for all six linear measurements were found by One‐way ANOVA (Table S4 and Figure S1). CV1 explained 59.77% of the variation, mainly representing the ratio of culmen length and wing length, and CV2 explained 23.79% of the variation, mainly representing the ratio of wing length, weight to body length and culmen (Table S5 and Figure S2). Although these two variable axes cannot distinguish all species, they can show a clear distribution trend (Figure S2a), and there is a significant Mahalanobis distance between each pair of species (Table S5).

**FIGURE 2:**
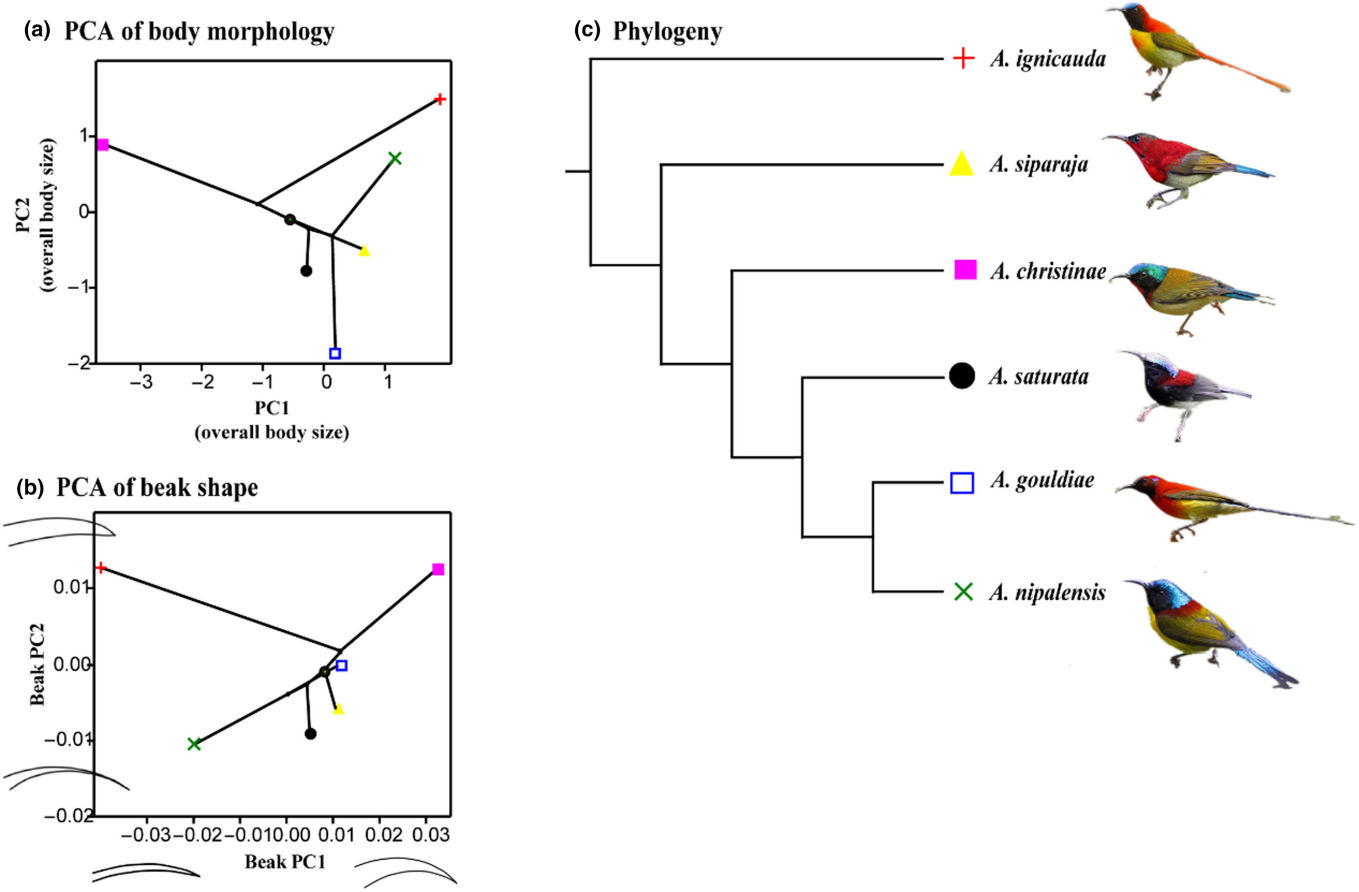
Scatters of the principal component analysis of the six species of *Aethopyga*. (a) projection pf phylogeny onto shape space of body morphology (PC1 explained 62.16% of the morphological variation, representing the growth of all indicators. PC2 explains 25.68% of the total variation and mainly represents changes in the ratio of the body weight, culmen and tarsal length to body length, tail length and wing length); (b) projection pf phylogeny onto shape space of beak shape (PC1 (60.33%) represents the variation of the beak shape from a slender and straight to a thick and decurved beak, PC2 (19.71%) represents the shape variation from a blunt to a pointed beak); (c) the reconstructed phylogeny of the six species of *Aethopyga* birds used in this study. The species illustrations refer to HBW alive: http://www.hbw.com/.

Principal component analysis extracted the dimension with the largest body shape variation (Table S6). PC1 explained 62.16% of the morphological variation, representing the growth of all indicators, and can be interpreted as an indicator of overall body size difference. PC2 explains 25.68% of the total variation and mainly represents changes in the ratio of the body weight, culmen and tarsal length to body length, tail length, and wing length (Figure 2a). The scatter plot of PCs extracted from body measurements illustrates that PC1 could be used to distinguish *A. christinae* from other species in the morphospace, whereas the remaining species demonstrate a relatively similar morphology and are more concentrated in the morphological space (Figure 2a).

### 3.2 Variation in beak shape

Significant interspecific differences were found in both shape (*F* = 34.40, *p* < .0001) and size (*F* = 23.94, *p* < .0001) aspects of beak by the Procrustes ANOVA. The canonical variable analysis extracted the dimensions with the largest interspecific beak shape differences. CV1 explains 66.78% of the total variation and is interpreted mainly as changes in beak shape from slender and straight to thick and curved; CV2 explains 16.27% of the total variation, which represents the variation from blunt to pointed shape of the beak and the relative position of the upper and lower beak endpoints; CV3 explained 6.81% of the total variation, representing the change from slender and curved to straight and stubby (Figure S2d). The scatter plot of CVs extracted from beak configurations shows that CV1 and CV2 could be used to distinguish *A. ignicauda* from other species, while the remaining species overlap to a large extent in the morphospace (Figure S2c). However, the results show that there is a significant Mahalanobis distance between most pairs of species (Table S7).

**FIGURE 3:**
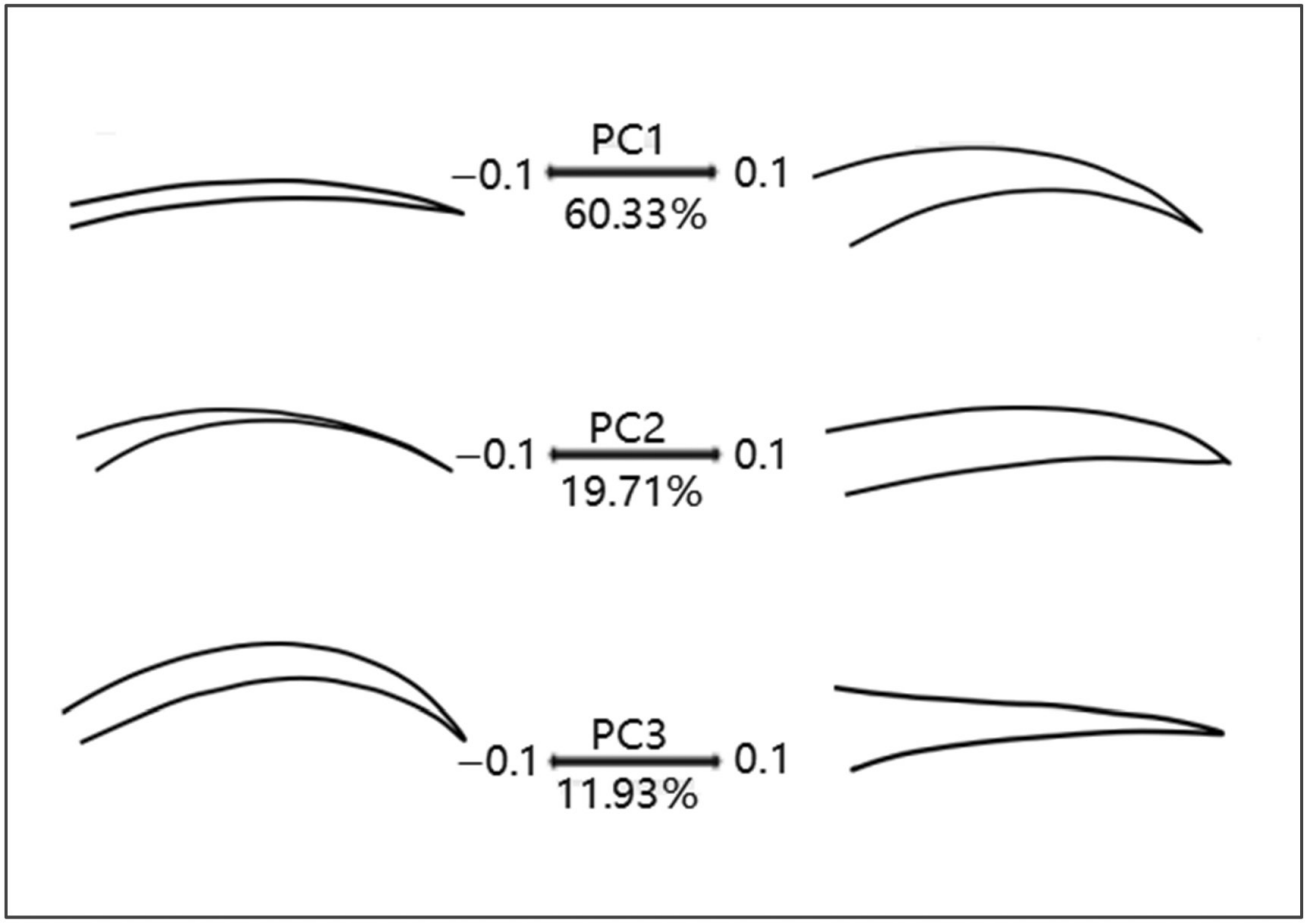
Patterns of shape change associated with PCs calculated from beak shape variation.

The Procrustes PCA suggest that over 91.97% of the total variance was concentrated in the first three axes (Figure 3). PC1 represents the variation of the beak shape from a slender and straight to a thick and decurved beak, PC2 represents the shape variation from a blunt to a pointed beak, and PC3 represents changes in the curvature of the beak (Figure 3). The scatter plot of PC1 and PC2 shows that *A. gouldiae*, *A. saturata*, and *A. siparaja* are concentrated in morphological space, while *A. nipalensis*, *A. ignicauda*, and *A. christinae* are seen distinguishable in the main cluster (Figure 2a).

### 3.3 Evolutionary changes and phylogenetic signals

In this study, the phylogenetic tree was projected into the morphological space (Figure 2). The scatter plot of PC1 and PC2 extracted from either body morphology or beak shape shows no visible clustering of closely related species in the morphospace. There are great morphological differences between birds and their closest relatives, and a large number of crossing branches and long branches that are disproportionate to the evolution time. The results of phylogenetic signal detection showed no clear significant phylogenetic signal (*p* > .05) in the morphological characteristics of these six species (Table S8).

### 3.4 Covariation between morphology and altitude

**FIGURE 4:**
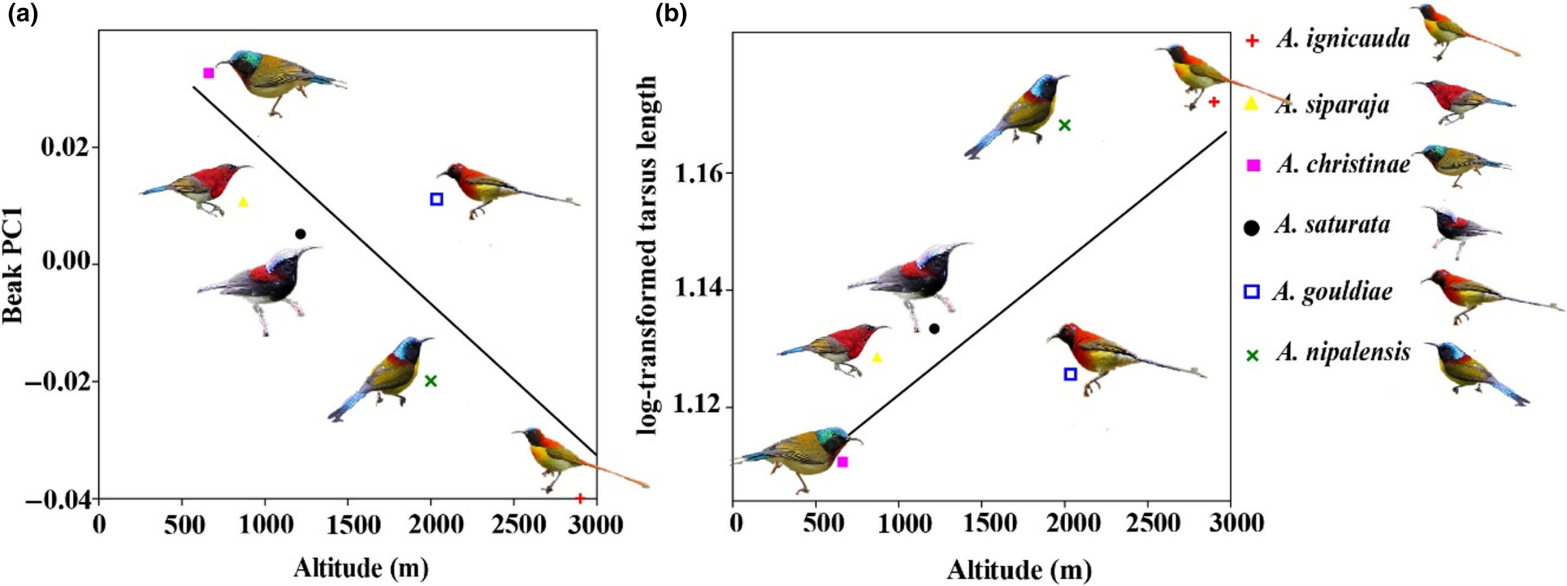
Interspecific covariation between morphological traits and altitude of the six species examined. Correlations of altitude with tarsus and PC1 of beak shape are shown. Dots with different colors and shapes represent different species. Lines represent phylogenetic generalized least square (PGLS) regressions.

**FIGURE 5:**
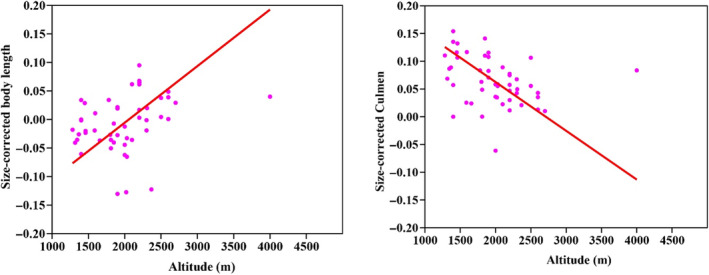
Intraspecific covariation between morphological traits and altitude within *Aethopyga nipalensis*. Correlations of altitude with body length and Culmen. Each dot represents an individual *A. nipalensis* specimen. Lines represent ordinary least square (OLS) regressions.

The results of the PGLS analysis show significant correlation of altitude with log‐transformed tarsus length (*beta* = 0.0000221, *p* = .0288) and beak‐shaped PC1 (slender/straight vs thick/decurved) (*beta* = −0.00002572, *p* = .0194) across the six species analyzed in this study (Figure 4 and Table S9). No significant altitudinal covariation was discovered for other morphological measurements. Within *A. nipalensis* individuals, altitude is correlated with multiple morphological characteristics (Figure 5 and Table S9). Individuals living at higher elevations typically exhibit longer body length (*R* ^2^ = .095801, *p* = .0384) but shorter culmen (*R* ^2^ = .12456, *p* = .0146) compared with those at lower elevations (Figure 5).

### 3.5 Character divergence and altitude distribution overlap

Since *A. christinae* and other species have less overlap, the Mantel test was performed on two datasets, one including all six species and one without *A. christinae*. The results of the partial Mantel test show that among six species, the distance matrix of body morphological characteristics did not show a significant correlation with the altitudinal distance matrix, while the distance matrix of beak morphological characteristics showed a significant positive correlation with the altitudinal distance matrix (altitude distribution) (Table S10). In terms of beak shape, species with greater overlap in elevation distribution have more similar morphological characteristics, that is, less morphological differentiation.

When *A. christinae* is excluded from the analysis, the results of the partial Mantel test showed that the distance matrix of body morphological characteristics and beak morphological characteristics did not show a significant correlation with the altitudinal distance matrix (Table S10).

## 4 DISCUSSION

This study employed traditional and geometric morphometrics to quantify the morphological variation of six *Aethopyga* species belonging to the main clades. Additionally, we investigated how morphological evolution is influenced by factors, such as phylogenetic relationship, altitudinal range, and species interactions.

We identified body morphological variation in two main axes: overall body size and body shape (relative larger body weight, longer culmen, and longer tarsal length and shorter body length, tail length, and wing length). Body weight of birds can be an indication of adaptation to environment temperature (Olson et al., 2009), as well as competition for better food resources (Temeles & Kress, 2003), while variation of body shape reflects interspecific difference in foraging mode and resource partitioning. *Aethopyga* sunbirds are considered to be mainly perching during nectar feeding (Janeček, 2011), which is associated with short tarsus (Zeffer et al., 2003). However, different species exhibit preference of different levels in the woods, from canopy down to understorey and sometimes on ground (del Hoyo et al., 2020), which may also exert selective pressure on hind limb. Some sunbird species can be quite aerial; for example, *A. ignicauda* has been observed to catch insects in flight (Cheke & Mann, 2020a, 2020b), corresponding to shorter wings and tail for maneuverability (Marchetti et al., 1995).

On the other hand, the variation of the beak shape of *Aethopyga* sunbirds is mainly concentrated in the dimensions from slender/straight vs thick/decurved. Bird beaks are closely relevant to diet and foraging behavior (Kulemeyer et al., 2009; Pigot et al., 2020). The variation of the beak shape of nectarivorous birds is considered to be the result of co‐adaptations with their preferred food. Co‐adaptations may occur in mutualistic systems, such as flowering plants and their pollinators, resulting in plants developing floral corolla shapes that match the feeding apparatus of their most efficient animal pollinators (e.g., Darwin & Bronn, 1862; Rothschild & Jordan, 1903; Temeles & Kress, 2003). Birds with short bills prefer flowers with short corollas, whereas birds with long bills can efficiently drink from both short and long flowers but often choose longer flowers because they provide larger amounts of nectar that is harder for short‐billed birds to extract (Janeček, 2011; Temeles & Kress, 2003). The significance of morphological co‐adaptations for the assembly of plant–pollinator interaction networks has been documented (Sazatornil et al., 2016; Soteras et al., 2018; Weinstein & Graham, 2017). Previous studies have demonstrated that the degree of species specialization in a given network is influenced by the morphological features of hummingbird species, particularly the shape and size of their beaks (Maglianesi et al., 2014). Despite that hummingbirds are more specialized in their interactions than *Aethopyga* sunbirds (Zanata et al., 2017), the variation of beak shape (slender/straight vs thick/decurved) in *Aethopyga* sunbirds, another group of nectar‐feeding birds, may also be attributed to trait matching. In addition, the pointed beak shape of *A. saturate* and *A. siparajarobs* can be related to their behavior of nectar‐robbing by piercing corolla bases of flowers (Cheke et al., 2020; Cheke & Mann, 2020a, 2020b). On the other hand, although *Aethopyga* sunbirds are often thought to be nectarivorous, their diet actually consists of a significant proportion of invertebrates (del Hoyo et al., 2020), which may also play a role in the variation of the beak shape. Furthermore, sexual selection may also play a role in the evolution of beak shape of *Aethopyga* species (Derryberry et al., 2018; Huber & Podos, 2006).

The phylogenetic signal test failed to find significant phylogenetic structure in any morphological characteristics of the six species. This may be partially due to the limited number of species analyzed and incomplete phylogeny used in this study, but this also provide indications that the morphological characteristics of *Aethopyga* broke away from the restriction of phylogenetic relations, probably due to selective pressure. Further study with a larger scale of *Aethopyga* species included will hopefully be able to capture a more precise correlation between phylogenetic relatedness and phenotypic similarity.

This study found that among the six *Aethopyga* species, the altitudinal distribution was negative correlated with PC1 of beak morphology (slender/straight vs thick/decurved dimension) and positive correlated with tarsus length. This result suggests that the altitude distribution does have a certain selective effect on the morphological characteristics of *Aethopyga* birds. The altitude distribution is related to the type of habitat, and then through food, foraging behavior, activity methods, it exerts selective pressure on the evolution of the form of organisms. The analysis of this study shows that species with thicker and especially curved bills were less common at higher elevations, where mostly species with slender and straight or slightly curved bills inhabited (López‐Segoviano et al., 2021; Maglianesi et al., 2015). Sonne et al. (2019) found that in Podopcarpus National Park, the longest corolla length were observed in highland regions, while the most pronounced corolla curvatures were found in lowland areas. Hořák and Janeček (2021) have shown that long‐billed sunbirds tend to adapt to the morphology of various Impatiens plant species in their local environment. Therefore, we can infer that the altitudinal pattern of beak variation in *Aethopyga* sunbirds (blunt and decurved vs slender and straight) might also be attributed to co‐adaptation with ornithophilous plants and geographical variation in trait matching. Another explanation is that with the increase of the altitude gradient, the shape of the beak changes from thick/decurved vs slender/straight, which may be related to the temperature of the environment in which it inhabits. In this study that the tarsus grows longer as the elevation gradient increases, perhaps because the evolution of larger feet enables *Aethopyga* sunbirds to perch while extracting nectar, which reduces the energy costs of hovering at high elevations. Derryberry et al. (2018) have demonstrated that body size and beak size exert different effects through their constraints on signal production and modification. For instance, in more open habitats, bird vocalizations tend to have higher speeds, while birds with larger beaks produce slower vocalizations. Therefore, body size and beak size may also be associated with song propagation.

The variation in size within a species along an altitudinal gradient may be a result of its response to changes in temperature (Ashton, 2002; Mayr, 1956), which is supported by our results that *A. nipalensis* individuals with longer body length dwells in higher altitudes. Our analysis also discovered a correlation between beak length and altitude, which can be attributed to a strategy of minimizing heat loss in high‐altitudinal cold environment (Greenberg & Danner, 2012). The morphological variation along the altitudinal gradient within a species further supported that altitudinal distribution exerts selective pressure on the phenotype of organisms and may drive divergent evolution.

Environment can pose selective pressure to a group of animals through the effect of environmental filtering or competition and resource partitioning. Environmental filtering typically leads to an increase in functional similarity among species within an assemblage (i.e., functional clustering), as it narrows down the range of trait values (Keddy, 1992). On the other hand, competition and resource partitioning usually lead to a limitation of functional similarity among co‐existing specie (i.e., functional over‐dispersion) (Macarthur & Levins, 1967; Mouchet et al., 2010). An example of this can be seen in the partitioning of nectar plants among different hummingbird species, which tend to become more specialized when there is high interspecific competition (Inouye, 1978; Schoener, 1974). In this study, some morphological characteristics of *Aethopyga* sunbirds showed correlation with altitudinal distribution, indicating that *Aethopyga* sunbirds at the same altitude have adapted to similar vegetation, habitat and food types. In addition, this study found that the beak shape of *Aethopyga* sunbirds was more similar in species with greater overlap in altitudinal distribution. The morphological convergence, associated with overlapping distributions, reflects the adaptation of sunbird species to similar selection pressures in similar environments. Therefore, we propose that environment filtering plays a more important role in the morphological evolution of the six sunbird species on a larger geographical scale, resulting in greater overlap of beak shapes among species with overlapping altitudinal distribution. On the other hand, locally coexisting *Aethopyga* sunbirds may diverge in microniches to avoid competition. Previous study (Li, 2019) has observed that co‐existing *Aethopyga* sunbirds tend to occupy different layers in the forest, and their diet contains approximately half of invertebrates and half of nectar from different *Rhododendron* species (*Rhododendron neriiflorum* for *A. gouldiae*, *R. neriiflorum* and *Rhododendron arizelum* for *A. ignicauda*, and *Rhododendron edgeworthii* for *A. nipalensis*). In other regions in Yunnan, *A. ignicauda* and *A. nipalensis* were found to choose other different species of *Rhododendron* as a food source (Huang et al., 2017). We speculate that the foraging preference and food choice are varied according to local condition and whether there are sympatric relatives, so as to partition resources and avoid competition, but these changes in strategy might not be reflected by morphological traits. Overall, our results suggest that altitudinal distribution may be critical for morphological variation in sunbird species, although we were unable to directly test how environmental filtering and the intensity of interspecific competition affect morphological traits.

One of the biggest limitations for this study was the lack of quantitative data on food characteristics. Detailed information about the network between *Aethopyga* sunbirds and ornithophilous plants should be collected and comparisons between their morphological traits should be incorporated to investigate whether there is coevolution. In addition, morphological and ecological data of more co‐existing populations at finer elevation scales are needed to further explore sympatric competition. Another limitation of this study is the scarcity of sunbird species. Further studies of morphological evolution and phylogenetic implications require the use of data from more *Aethopyga* species globally. Another limitation is that only male individuals were included in the analyses. Including individuals from both sexes would provide insights into various aspects. For instance, O'Connell et al. (2019) demonstrated a significant change in sexual dimorphism levels in sunbird populations on islands compared with the mainland. However, due to difficulties in obtaining an adequate sample size of female *Aethopyga* specimens, we focused on male samples to minimize errors. The primary objective of our study was to investigate interspecific differences and altitudinal selection.

## 5 CONCLUSIONS

In this study, we characterize the morphological traits of six *Aethopyga* species distributed in China. The main distinguishing characteristic among these six species are overall body size, the ratio of body and wing length to tarsus and culmen length, and beak shape (slender/straight vs thick/decurved). The results show that most species can be distinguished by differences in body shape and beak shape. The results of this study indicate that the morphological evolution of these six species of *Aethopyga* is not constrained by phylogenetic relatedness. Altitude is correlated with multiple morphological traits. Based on our findings, it is likely that altitude may be an ecological factor that exerts distinct selective pressures, potentially leading to the divergence of *Aethopyga* species. The morphological convergence related to distribution overlap reflects the adaptation to similar selection pressures in similar environments.

## AUTHOR CONTRIBUTIONS

**Wenzhu Lu:** Data curation (equal); formal analysis (equal); investigation (equal); methodology (equal); software (equal); writing – original draft (lead); writing – review and editing (equal). **Shimiao Shao:** Conceptualization (equal); formal analysis (equal); methodology (equal); resources (equal); software (equal); writing – original draft (equal); writing – review and editing (equal). **Lingling Zu:** Data curation (equal); investigation (equal); resources (equal). **Xu Luo:** Conceptualization (equal); data curation (equal); formal analysis (equal); supervision (equal); validation (equal); writing – original draft (equal). **Yubao Duan:** Conceptualization (lead); formal analysis (lead); funding acquisition (equal); methodology (lead); project administration (lead); resources (equal); supervision (equal); visualization (equal); writing – original draft (equal).

## FUNDING INFORMATION

This work was supported by the Science and Technology Plan Project of Yunnan Province (202101AT070040, 202301BD070001082 and 2019FD070) and the First Class Forestry Academic Subject in Yunnan Province. The funders had no role in study design, data collection and analysis, decision to publish, or preparation of the manuscript.

## CONFLICT OF INTEREST STATEMENT

The authors declare that they have no competing interests.

## Supporting information


Appendix S1

Appendix S2

Appendix S3

Appendix S4

Appendix S5

Data S1

## Data Availability

All data generated or analyzed during this study are included in this article. Further inquiries can be directed to the corresponding authors.
