# Supplementary material for: Morphological diversity and altitudinal differentiation of Aethopyga species
Source: Ecol Evol. 2023 Aug 30;13(9):e10473. doi: 10.1002/ece3.10473 (PMC10468329; doi:10.1002/ece3.10473)
Supplement: Supplementary file 1 — Appendix S1 [file ECE3-13-e10473-s001.pdf]

Additional File 1. Supplementary materials.

Table S1. List of Aethopyga species in this study.

Table S2. Methods for the linear morphological measurements.

Table S3. List and gene information of species in this study

Table S4. Summary statistics for body morphology of Aethopyga species examined.

Table S5 Mahalanobis distances for body morphology among species.

Table S6. Results of the PCA of body morphology.

Table S7. Mahalanobis distances for beak shape among species.

Table S8. Phylogenetic signals for each morphological character.

Table S9. Covariation between morphology and altitude.

Table S10. Results of the Mantel test for the correlation between character divergence and distributional distances.

Figure S1. Box plots of body characters of the 6 species of Aethopyga Sunbirds examined.

Figure S2. Results of the canonical variate analysis (CVA) on the morphology of individuals from the 6 species examined.

Table S1 list of *Aethopyga* species in this study

Common name	Scientific name	Altitude/m	Sample size	
			Body	Beak
Gould's Sunbird	*Aethopyga gouldiae*	1296-3975	81	81
Green-tailed sunbird	*Aethopyga nipalensis*	1282-4000	50	50
Fork-tailed Sunbird	*Aethopyga christinae*	140-850	25	25
Black-throated Sunbird	*Aethopyga saturata*	300-2207	56	56
Crimson Sunbird	*Aethopyga siparaja*	500-2978	63	63
Fire-tailed Sunbird	*Aethopyga ignicauda*	1460-3300	23	23
Total		140-4000	298	298

Table S2. Methods for the linear morphological measurements.

Character	Method
Body weight	Weighed in the field by collectors.
Body length	Measured in the field by collectors.
Wing length	The distance between the bend of wing to the tip of longest primary, measured on the closed wing.
Tail length	The distance from the root of central pairs of tail-feathers to tip of longest tail-feather, measured when the tail is naturally folded.
Tarsus length	The distance from the notch on the back of the inter-tarsal joint to the lower edge of the last complete scale before the toes diverge.
Culmen	The distance from the tip of the bill to the distal edge of the nostrils.

References:
Svenson L, Identification guide to European Passerines; London; British Trust for Ornithology; 1992.

Table S3 List and gene information of species in this study

Taxon	English name	Catalogue no.
Aethopyga bella	Handsome sunbird	KU 19341
Aethopyga bella	Handsome sunbird	KU 19631
Aethopyga boltoni boltoni	Apo sunbird	CMNH 1816
Aethopyga boltoni boltoni	Apo sunbird	CMNH 1880
Aethopyga boltoni tibolii	Apo sunbird	CMNH 2197
Aethopyga boltoni tibolii	Apo sunbird	CMNH 2222
Aethopyga boltoni tibolii	Apo sunbird	CMNH B36477
Aethopyga christinae	Fork-tailed sunbird	KU 13901
Aethopyga flagrans	Flaming sunbird	KU 18055
Aethopyga flagrans	Flaming sunbird	KU 19598
Aethopyga flagrans guimarasensis	Flaming sunbird	KU 15352
Aethopyga flagrans guimarasensis	Flaming sunbird	KU 15802
Aethopyga gouldiae	Gould's sunbird	KU 11048
Aethopyga gouldiae	Gould's sunbird	KU 11236
Aethopyga ignicauda	Fire-tailed sunbird	KU 5649
Aethopyga ignicauda	Fire-tailed sunbird	KU 15233
Aethopyga linarabora	Lina's sunbird KU	35806
Aethopyga linarabora	Lina's sunbird KU	35868
Aethopyga linarabora	Lina's sunbird KU	B35869
Aethopyga nipalensis	Green-tailed sunbird	KU 5549
Aethopyga nipalensis	Green-tailed sunbird	KU 15234
Aethopyga primigenia	Grey-hooded sunbird	KU 19100
Aethopyga pulcherrima pulcherrima	Metallic-winged sunbird	KU 19024
Aethopyga pulcherrima jeffreyi	Metallic-winged sunbird	KU 19654
Aethopyga pulcherrima decorosa	Metallic-winged sunbird	KU 20924
Aethopyga saturata	Black-throated sunbird	AMNH DOT2630
Aethopyga saturate	Black-throated sunbird	AMNH DOT10779
Aethopyga shelleyi	Lovely sunbird	KU 12723
Aethopyga shelleyi	Lovely sunbird	KU 12777
Aethopyga siparaja siparaja	Crimson sunbird	AMNH DOT331
Aethopyga siparaja seheriae	Crimson sunbird	AMNH DOT12310
Aethopyga siparaja	Crimson sunbird	AMNH23132
Aethopyga siparaja magnifica	Crimson sunbird	KU 15302
Aethopyga temminckii	Temminck's sunbird	KU 17752
Anthreptes malaccensisd	Brown-throated sunbird	KU 14049

References:

Hosner P A, Nyári Á S, Moyle R G. Water barriers and intra-island isolation contribute to diversification in the insular *Aethopyga* sunbirds (A ves: N ectariniidae)[J]. Journal of Biogeography, 2013, 40(6): 1094-1106.

Table S4. Summary statistics for body morphology of *Aethopyga* species examined.

Species	N	Body weight	Body length	Wing length	Tail length	Tarsus length	Culmen
Aethopyga gouldiae	81	6.12±0.13	132.16±1.30	54.47±0.19	65.59±1.27	13.35±0.11	14.35±0.09
Aethopyga nipalensis	50	6.60±0.10	125.21±1.46	52.65±0.35	58.31±1.04	14.70±0.11	19.28±0.23
Aethopyga christinae	25	5.97±0.12	100.95±4.02	48.08±0.45	36.07±1.65	12.90±0.25	14.27±0.26
Aethopyga saturata	56	5.85±0.14	122.05±3.38	51.31±0.28	60.96±1.45	13.59±0.12	16.61±0.15
Aethopyga siparaja	63	7.02±0.11	127.95±1.20	54.78±0.21	58.92±1.08	13.44±0.08	16.80±0.10
Aethopyga ignicauda	23	8.58±0.17	127.13±4.33	54.73±0.52	52.34±4.09	14.86±0.23	18.12±0.21
Total	298	6.52±0.07	125.17±1.04	53.12±0.16	58.57±0.77	13.72±0.06	16.39±0.12
F		97.5	15.01	57.9	27.28	23.87	153.7
p		< 0.0001	< 0.0001	< 0.0001	< 0.0001	< 0.0001	< 0.0001

The value of each characteristic is given as mean ± SE.

Table S5 Mahalanobis distances for body morphology among species.

Species	*A. nipalensis*	*A. ignicauda*	*A. saturata*	*A. siparaja*	*A. gouldiae*	*A. christinae*
A. nipalensis						
A. ignicauda	8.07127					
A. saturata	5.68603	14.3358				
A. siparaja	7.0723	6.36792	4.21398			
A. gouldiae	22.6985	20.3678	9.10353	6.57765		
A. christinae	26.0534	26.0917	12.002	16.6639	14.6678	

Table S6. Results of the PCA of body morphology.

	Eigenvalue	% variance	Loading coefficients of morphological characteristics					
			Body weight	Body length	Wing length	Tail length	Tarsus length	Culmen length
PC1	3.72973	62.162	0.3447	0.4505	0.4637	0.3781	0.4156	0.3842
PC2	1.5409	25.682	0.4488	-0.3926	-0.2147	-0.5264	0.4136	0.3875
PC3	0.624487	10.408	-0.6242	-0.00113	-0.4283	0.2519	0.2685	0.5398

Table S7. Mahalanobis distances for beak shape among species.

Species	*A. nipalensis*	*A. ignicauda*	*A. saturata*	*A. siparaja*	*A. gouldiae*	*A. christinae*
A. nipalensis						
A. ignicauda	*4.94E-13*					
A. saturata	*2.86E-17*	*6.24E-20*				
A. siparaja	*1.95E-21*	*2.09E-12*	*9.77E-16*			
A. gouldiae	*1.02E-47*	*7.07E-30*	*8.08E-31*	*1.22E-26*		
A. christinae	*1.11E-26*	*9.74E-17*	*7.95E-19*	*2.52E-24*	*5.67E-26*	

Table S8. Phylogenetic signals for each morphological character.

Character	6 species			
	K	P-value	λ	P-value
Log10(Body weight)	1.3804	0.052	0.0001	1.000
Log10(Body length)	0.3471	0.611	0.9999	0.792
Log10(Wing length)	0.3961	0.475	0.0001	1.000
Log10(Tail length)	0.3379	0.619	0.9999	0.643
Log10(Tarsus length)	0.3657	0.58	0.0001	1.000
Log10(Culmen length)	0.2318	0.939	0.0001	1.000
Size-corrected body length	0.5003	0.220	0.0001	1.000
Size-corrected wing length	0.4014	0.472	0.0001	1.000
Size-corrected tail length	0.6606	0.077	0.9501	0.737
Size-corrected tarsus length	0.3169	0.690	0.0001	1.000
Size-corrected culmen length	0.2954	0.829	0.0001	1.000
PC1of body morphology	0.4236	0.482	0.0001	1.000
PC2 of body morphology	0.3094	0.754	0.0001	1.000
PC1 of beak shape	0.5559	0.214	0.0001	1.000
PC2 of beak shape	0.4452	0.316	0.0001	1.000

Table S9. Covariation between morphology and altitude.

Character	PGLS of species means		Individuals within *A. nipalensis*	
	Beta	P-value	R2	P-value
Log10(Body weight)	0.0000546	0.0888	0.045319	0.1701
Log10(Body length)	0.0000253	0.2564	0.076427	0.0583
Log10(Wing length)	0.0000177	0.1777	0.005702	0.6207
Log10(Tail length)	0.0000425	0.2503	0.029624	0.2453
Log10(Tarsus length)	0.0000221	**0.0288***	0.016709	0.4024
Log10(Culmen length)	0.0000249	0.3273	0.3227	**0.0001***
Size-corrected body length	-0.000007158	0.7878	0.095801	**0.0384***
Size-corrected wing length	-2.0645E-06	0.8663	0.036814	0.2005
Size-corrected tail length	-0.0000372	0.6064	0.0061609	0.5992
Size-corrected tarsus length	0.000002852	0.8078	0.06694	0.0873
Size-corrected culmen length	-0.000012845	0.6715	0.12456	**0.0146***
PC1of body morphology	0.0017692	0.0947		
PC2 of body morphology	0.0003439	0.6506		
PC1 of beak shape	-0.00002572	**0.0194***		
PC2 of beak shape	0.000002317	0.7229		

"*" indicates statistical significance ($P < 0.05$).

Table S10. Results of the Mantel test for the correlation between character divergence and distributional distances.

Dataset	Distribution	Body morphology		Beak shape	
		R	P-value	R	P-value
6 species	Altitudinal	0.3515107	0.1218878	0.3688632	0.1681832
5 species	Altitudinal	-0.2261746	0.2252775	0.361789	0.105179
Partial mantel	Altitudinal				
6 species	Altitudinal	0.3539881	0.1285871	0.64817408	**0.04419558***
5 species	Altitudinal	-0.26118	0.215957	0.4552909	0.082092

"*" indicates statistical significance ($P < 0.05$).

Figure S1

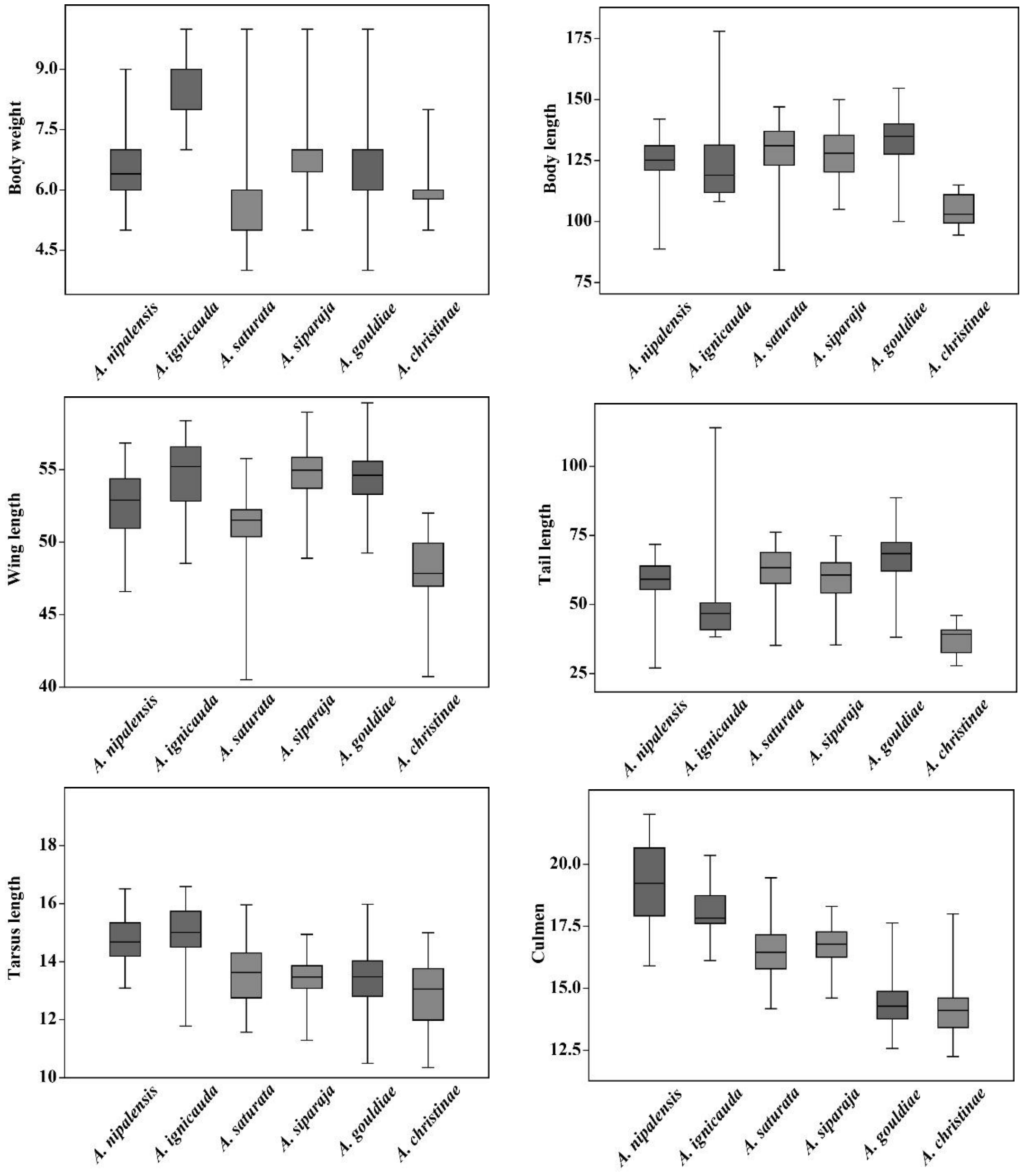

Figure 1 Box plots of morphological characters of the 6 species of *Aethopyga* Sunbirds.

Figure S2

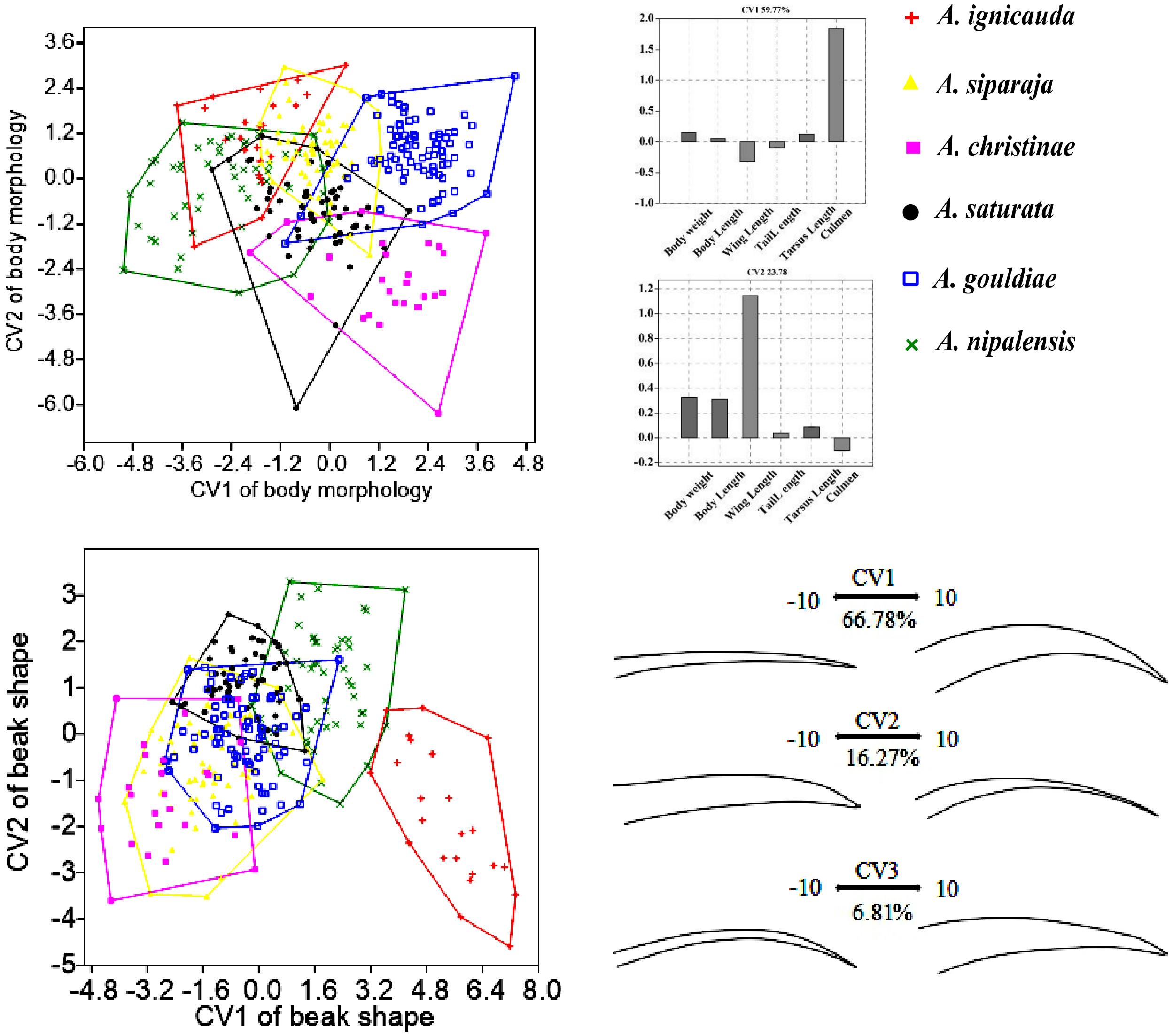

Figure 2 Results of the canonical variate analysis on the morphology of the 6 species of *Aethopyga* Sunbirds. A: Plot of CV1 and CV2 for body morphology; B: Loading coefficients of CV1 and CV2; C: Plot of CV1 and CV2 for beak shape; D: Patterns of beak shape change associated with CVs.
