# Supplementary material for: Morphological diversity and altitudinal differentiation of Aethopyga species
Source: Ecol Evol. 2023 Aug 30;13(9):e10473. doi: 10.1002/ece3.10473 (PMC10468329; doi:10.1002/ece3.10473)
Supplement: Supplementary file 6 — Data S1 [file ECE3-13-e10473-s006.docx]

**Supplementary Material**

**Additional file 1:** Supplementary materials. Included are 10 supplementary tables (Table S1-S10) and 2 supplementary figures (Figures S1-S2). (PDF 531 kb).

**Additional file 2:** Body measurements. Data for the linear measurements of all specimens involved in this study are shown, including body weight, body length, wing length, tail length, tarsus length and culmen. The altitude for each specimen is also included.(CSV 24 kb).

**Additional file 3:** Coordinates of landmarks for geometric morphometric analysis of beak shape. (CVS 105 kb)

**Additional file 4:** Phylogeny of the 6*Aethopyga* species analysed in this study. (word 13 kb)

**Additional file 5:** R script (4kb).
